# Effects of knee flexion and extension on the tibial tuberosity–trochlear groove (TT–TG) distance in adolescents

**DOI:** 10.1186/s40634-018-0149-1

**Published:** 2018-08-16

**Authors:** Juha-Sampo Suomalainen, Gideon Regalado, Antti Joukainen, Tommi Kääriäinen, Mervi Könönen, Hannu Manninen, Petri Sipola, Hannu Kokki

**Affiliations:** 1 Department of Clinical Radiology, Kuopio University Hospital, Kuopio, Finland; 2 Department of Orthopaedics and Traumatology, Kuopio University Hospital, Kuopio, Finland; 3 School of Medicine, University of Eastern Finland, P.O. BOX 100, FI-70029 KYS Kuopio, Finland

**Keywords:** Kinematic malalignment, Patellofemoral instability, Patellar dislocation, Tibial tubercle–trochlear groove distance, Magnetic resonance imaging

## Abstract

**Background:**

Measurement of the tibial tubercle–trochlear groove (TT–TG) distance is used to assess patellofemoral instability and rotation. Since patellofemoral instability and acute patellar dislocation are common among adolescents, it is important to clarify the relationship between TT–TG distance and various flexion and extension angles in asymptomatic children. The purpose of the present study was to determine how knee flexion and extension influence TT–TG-distance values measured using 3D imaging in an anatomic axial plane among asymptomatic adolescents.

**Methods:**

We performed magnetic resonance imaging (MRI) of 26 knees in 13 adolescents (8 boys and 5 girls) of 11–17 years of age, with no known patellofemoral disorders. Imaging was performed with 3.0 T MRI with the knee at four separate angles of flexion between 0° and 30°. Measurements were made by two independent blinded raters.

**Results:**

The mean TT–TG distance in millimetres was 11.1–0.29 × the angle in degrees. TT–TG distance decreased with greater flexion, showing a mean decrease of 0.29 mm (SD, 0.04) per degree of increased flexion (*p* < 0.001). We found significant inter-observer (Pearson’s *r* = 0.636, *p* = 0.03) and intra-observer (Pearson’s *r* = 0.792, *p* ≤ 0.001) correlations. TT–TG values were not significantly correlated with age, length, weight, or body mass index. The rate of TT–TG change (change between consecutive TT–TG values/change between consecutive angles) was significantly negatively correlated with length (*p* = 0.014), weight (*p* = 0.004), and body mass index (*p* = 0.025).

**Conclusions:**

Our data revealed that TT–TG distance assessed in the anatomic axial plane decreased with greater flexion in adolescent. Moreover, this effect of knee angle was stronger in smaller subjects. These findings support the need for a standardized protocol for TT–TG distance measurement in adolescents.

## Background

Patellofemoral instability and acute patellar dislocation are common among adolescents, and acute patellar dislocation occurs with an annual incidence of 43/100,000 in children under 16 years of age (Ferlic et al. 2018). The main predisposing factors for patellar instability include trochlear dysplasia, lateral patellar inclination, patella alta, and increased lateral quadriceps vector (Arendt and Dejour 2013; Dejour et al. 1994; Nietosvaara et al. 1994). Patellar instability among adolescents is increasingly quantified based on tibial tubercle–trochlear groove (TT–TG) distance. Several studies have described the use of TT-TG in adults (Camathias et al. 2016; Dietrich et al. 2014; Izadpanah et al. 2014; Tanaka et al. 2015), but relatively little data are available regarding TT–TG values in healthy children (Dickens et al. 2014).

TT–TG distance measured on computed tomography images is considered the gold standard for assessing the increased lateral quadriceps vector (Hinckel et al. 2015a). However, it has been proposed that TT–TG can also be reliably determined by magnetic resonance imaging (MRI) using either cartilage or bony landmarks (Schoettle et al. 2006), but data indicates that normal values of TT-TG obtained with CT scan and MRI are not the same (Hinckel et al. 2015b; Schoettle et al. 2006).

There remains a need for an appropriate protocol for measuring TT–TG distance, especially in children and adolescents, as it can be substantially influenced by minor alterations in the axial scan plane orientation. For example, a mere 5° relative femoral abduction or adduction is associated with mean changes of ~ 40% in TT–TG distance (Yao et al. 2014). Indeed, TT–TG measurement in adults is invariably affected by knee flexion alone (Seitlinger et al. 2014).

To our knowledge, no prior study has evaluated how knee flexion influences TT–TG distance in asymptomatic adolescents. Therefore, our present study was designed to assess the effects of knee flexion on TT– TG values determined at different flexion-extension angles using 3D MRI in asymptomatic adolescents. The anatomic axial plane for TT–TG measurements was reconstructed from 3D data. The presently reported data may help in the evaluation of affected knees.

## Methods

For this study, we recruited 13 adolescent participants of 11–17 years of age, between December 2013 and January 2014. Subjects were children of the staff of Kuopio University Hospital, Kuopio, Finland, where the study was performed. Exclusion criteria were known patellofemoral disorders, previous history of knee pain, rheumatic disorders, instability, valgus or varus deformities upon clinical examination, having undergone any surgery on either knee, contraindications to MRI, and any signs of trochlear dysplasia that could alter the results (Daynes et al. 2016; Longo et al. 2016).

The included participants comprised 8 boys and 5 girls (a total of 26 knees). Participants had a median age of 14 years (range, 11–17 years). Each participant had an age- and sex-adjusted body mass index (ISO-BMI) of between 17.2 and 27.1 kg/m^2^ (median, 19.9 kg/m^2^) (Saari et al. 2011) (Table 1).

**Table 1:** Baseline characteristics. Data are median [minimum, maximum] or number of cases. ISO-BMI, International Obesity Task Force extension for body mass index to be used in children aged 2–18 years (Saari et al. 2011)

Sex (female/male)	5/8
Age (yrs)	14 [11, 17]
Height (cm)	170 [145, 186]
Weight (kg)	57 [33, 84]
ISO-BMI	19.7 [17.2, 27.1]

### Imaging

Examinations were performed using a 3.0 Tesla MRI scanner (Phillips Achieva 3.0 T; DA Best, the Netherlands) with a body coil to allow flexion-extension movement of the knee. Imaging was performed with the subjects in the supine position, having their knee on a popliteal support allowing a relaxed flexion of 30–40°. The adolescents were asked to actively extend their knee against gravity in four steps at 30°, 20°, 10° and 0° of flexion-extension, and to hold their knee still for imaging at each step. Appropriate knee flexion was assessed with the goniometry with the centre over the lateral epicondyle of femur, the proximal arm towards greater trochanter and the distal arm towards lateral malleolus. The selected angles of between 0° to 30° covered the typical knee angles imaged in routine practice. During analysis, each true extension-flexion angle was measured from the MR images as the angle between the axes of the distal femur and proximal tibia in the sagittal plane.

For 3D scanning, we used the gradient echo scout sequence: repetition time, 7.56 ms; echo time, 2.12 ms; continuous axial slices with a 2.4-mm thickness and 1.2-mm spacing between slices; matrix, 256 × 256; pixel size, 1.17 × 1.17; acquisition time, 1 min 18 s; and reconstructed slice thickness, 1.2 mm. To obtain TT–TG measurements, we generated 3D multiplanar reconstructions (MPR) using the MPR tool of the picture archiving and communication system (PACS; Sectra AB, Linköping, Sweden), from 3-mm-thick sagittal and axial slices with no gaps between slices. For axial slices, the anatomic axial plane was used - ie, the slices were parallel to the knee joint (Yao et al. 2014). On the other hand, the sagittal slices were positioned perpendicular to a line drawn along the posterior aspect of the femoral condyles.

Two experienced musculoskeletal radiologists (J-SS and PS) digitally measured the TT–TG parameter on T1-weighted anatomic axial MR images in the PACS workstation. J-SS took measurements from images of all of the subjects at all four angles (*N* = 104). To assess the extra-observer and intra-observer agreement, 20 of these measurements were performed again by both J-SS and PS (*N* = 20).

**Fig. 1:**
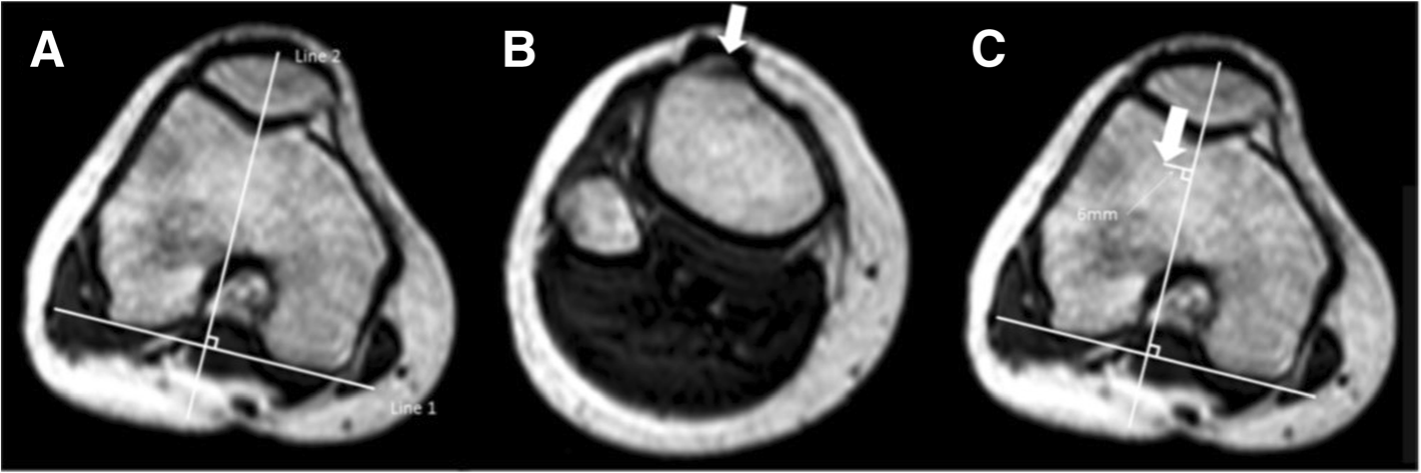
TT-TG distance is measured using axial slices. **a** First tangential line to the posterior condyles is drawn (Line 1). Next a line, perpendicular to Line 1, transecting the deepest point of the bony outline of the trochlear groove. This line is drawn to the most cranial slice with complete cartilaginous coverage of the trochlea (Line 2). **b** The most anterior point of the tibial tuberosity is defined as reference point. Keeping the cursor steadily in locations of the reference point, the image slab is then rolled back until the previously defined Line 2 is present. **c** The distance between the location of the cursor and Line 2 is measured in millimeters presenting the TT-TG distance, in this case 6 mm

**Fig. 2:**
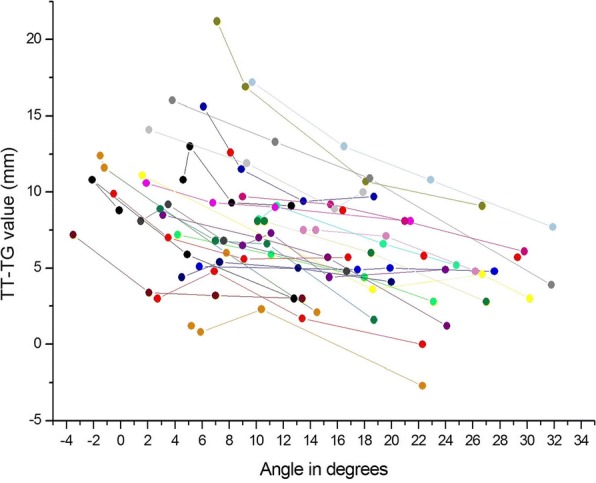
Individual TT–TG values in different knee flexion angles measured from magnetic resonance images

The TT–TG distance was measured using bony landmarks (Schoettle et al. 2006). We assessed the bony TT–TG between the most anterior point of the tibial tuberosity and the deepest bony point of the trochlear groove, perpendicular to the tangent to the bony borders of the posterior condyles on axial MRI scans. We first drew a line tangent to the posterior condyles (Goutallier et al. 1978). Next, this line was connected with the perpendicular line transecting the deepest point of the bony outline of the trochlear groove on the most cranial femoral sequence with complete cartilaginous coverage of the trochlea (Fig. 1). The image was rolled down to reveal the most anterior point of the tibial tuberosity, which was marked with the cursor as the first point (Fig. 2). With the cursor steadily kept in place, the image was then rolled back to reveal the previous markings. The TT–TG distance was then measured in millimetres, parallel to the posterior condylar line, between the first point and the line crossing the deepest point of the bony outline of the trochlear groove (Schoettle et al. 2006).

### Statistical analysis

Data were analysed using SPSS Statistics software, version 22.0 (International Business Machines Corporation, Armonk, NY, USA). The inter-observer and intra-observer correlations were determined based on Pearson’s correlations. We tested the effects of leg (left or right), angle, age, length, weight, and BMI on TT–TG value using mixed model analysis with a 95% confidence interval (CI). To analyse the rate of change of TT–TG, we calculated an additional variable (RATE) for consecutive angles using the following equation: RATE = change between consecutive TT–TG values/change between consecutive angles. We additionally analyzed the effects of age, length, weight, and BMI on this RATE value using mixed model analysis with 95% CI. Data are presented as mean with standard deviation (SD). A two-sided *p*-value of less than 0.05 was considered statistically significant.

## Results

The knee flexion angles were measured from sagittal MRI slices (Table 2). There was no problem to find out the trochlear groove and not subjects had dysplasia. Statistical analyses revealed a significant correlation between the two raters (Pearson’s *r* = 0.636, *p* = 0.03), as well as a significant intra-observer correlation (Pearson’s *r* = 0.792, *p* < 0.001).

**Table 2:** The measured knee angles. The knees were scanned in four different angles of flexion-extension. The angle was measured from the MR images as the angle between the axes of the distal femur and proximal tibia in the sagittal plane

Subject	Right Leg				Left Leg			
	Angle1	Angle2	Angle3	Angle4	Angle1	Angle2	Angle3	Angle4
Subj. 1	5.1	4.6	8.2	12.6	6.1	8.9	13.5	18.7
Subj. 2	2.7	6.9	13.4	22.3	10.4	5.2	5.9	22.3
Subj. 3	4.2	11.1	18.0	23.1	9.0	11.1	15.4	24.0
Subj. 4	5.8	17.5	19.9	27.6	9.0	15.5	21.0	29.8
Subj. 5	10.2	11.5	19.4	24.8	8.1	16.4	22.4	29.3
Subj. 6	1.9	6.8	11.4	21.4	2.1	9.3	15.8	17.9
Subj. 7	1.6	18.6	26.7	30.2	3.8	11.4	18.4	31.8
Subj. 8	7.1	9.2	18.1	26.7	10.6	10.1	18.5	27.0
Subj. 9	4.5	7.3	13.1	20.0	9.7	16.5	22.9	31.9
Subj. 10	3.1	10.2	15.3	24.1	13.5	14.4	19.6	26.2
Subj. 11	−3.5	2.1	7.0	13.4	1.5	3.5	7.6	16.7
Subj. 12	−1.5	−1.2	7.8	14.5	−0.1	−2.1	4.9	12.8
Subj. 13	2.9	7.0	10.8	18.7	−0.5	3.5	9.1	16.8

Examination of data models revealed similar goodness of fit for both the linear and logarithmic models, with correlations of the fitted values with the TT–TG and logarithmic TT–TG measurements of *r* = 0.944 and *r* = 0.915, respectively. We chose to use the linear model for further analysis in accordance with a prior study (Seitlinger et al. 2014).

The TT–TG distance decreased by 0.29 mm (SD, 0.04) per one degree of increased flexion (p < 0.001). The mean TT–TG distance in millimetres was 11.1–0.29 × the angle in degrees. TT–TG values were not significantly correlated with age, length, weight, or BMI. On the other hand, the rate of TT–TG change was significantly negatively correlated with length (*r* = − 0.016, *p* = 0.014), weight (*r* = − 0.015, *p* = 0.004), and BMI (*r* = − 0.059, *p* = 0.025), i.e. angle had a greater effect on TT–TG value in smaller subjects.

## Discussion

Patellar instability is a multifactorial condition (Feller et al. 2007), and proper treatment requires accounting for all of the static and dynamic factors that contribute to patellofemoral stability (Longo et al. 2016). TT–TG distance is a widely used parameter for assessing patellofemoral instability. Along with other factors, TT–TG measurement can help a clinician determine whether surgical intervention is required and whether medial patellofemoral ligament (MPFL) reconstruction alone is likely sufficient or if it should be combined with bony procedures, such as tibial tuberosity osteotomy or trochleoplasty. However, there are presently no absolute threshold values that clearly indicate the need for MPFL reconstruction combined with bony procedures (Longo et al. 2016). The uncertainties surrounding this topic increase the importance of understanding the factors that influence radiologic TT–TG assessment.

Our data indicated that TT–TG measurement was affected by knee flexion-extension in asymptomatic adolescents. Earlier data indicated that TT–TG distance of ≥15 mm is associated with greater probability of patellar instability (Schoettle et al. 2006). TT–TG distances of 15–20 mm were considered borderline, while a measurement of > 20 mm were considered to represent an excessive lateral position of the tuberosity. Our present findings in healthy adolescents demonstrated that no borderline TT–TG distances were measured when knee flexion was ≥10°, indicating that excess knee flexion can cause false-negative results. This is consistent with other studies showing that cut-off values may not be appropriate in evaluation of patellofemoral instability as TT-TG is related to the size of the knee and the patient (Ferlic et al. 2018; Hernigou et al. 2018; Hingelbaum et al. 2014).

We further demonstrated that the rate of TT–TG change was significantly negatively correlated with length, weight, and BMI. In other words, the effect of knee angle was greater in smaller subjects. This finding is particularly relevant since patellar instability is common among adolescents. Our present data indicate a need to standardize the TT–TG measurement protocol to avoid overlooking pathology, especially in adolescents. We suggest that the knee should be fully extended for imaging. Moreover, quadriceps should be at rest as the muscle contraction may impact TT-TG measures. If the knee is not fully extended this should be taken in account. Data in adults indicate that as the knee is flexed the TG deviates laterally in relation to the starting point when the knee is in extension (Iranpour et al. 2010). Rotation of the knee should be taken in account also. In the present study there was no problem to find out the groove because study subjects were healthy children, not patients with dysplasia.

Geometric accuracy and stability are critical for quantitative analysis from images. To avoid artefact, we used a brief sequence for MRI imaging, which generated images of a resolution more suitable for bony TT–TG measurement (Schoettle et al. 2006). In routine practice, the limb is often at rest, supported by the table, thus reducing artefact and potentially enabling longer sequences and better resolution. Such sequences might be conducive to the use of soft tissue landmarks, which are the true mechanical points of interest. However, most adolescents are normally hyperactive and not acquiescent to lying still in place for a longer period of time. Positioning an adolescent in a semi-enclosed sliding tube could pose a challenge to technicians. A technician must have time and patience to give clear instructions to the subject and ensure adequate positioning. Essential advice for imaging is to use the anatomic axial plane, and to thereby avoid errors caused by variances in extremity positioning along the plane of the table.

In this study, the results produced using the linear and exponential models of analysis were highly similar, possibly due to the limited number of subjects, that was the main limitation of this study. Our data are insufficient to determine whether knee flexion-extension has a linear or exponential effect on TT–TG-measurement values. Further studies are needed to explore this subject.

## Conclusion

The main finding of our present study was that the TT–TG distance decreased with increased flexion, which may give a false impression of normality from an image of a flexed knee. Moreover, this effect of knee angle was greater in smaller subjects. These findings indicate the importance of standardized knee flexion in a standardized TT–TG distance measurement protocol, especially for adolescent patients.

## Abbreviations

ISO-BMI: Age- and sex adjusted body mass index
MPFL: Medial patellofemoral ligament
MPR: Multiplanar reconstruction
MRI: Magnetic resonance imaging
RATE: Change between consecutive TT–TG values/change between consecutive angles
TT-TG: Tibial tubercle–trochlear groove
